# Development and validation of a chromatin regulator prognostic signature in colon adenocarcinoma

**DOI:** 10.3389/fgene.2022.986325

**Published:** 2022-11-23

**Authors:** Wenlong Yang, Chenhua Luo, Shan Chen

**Affiliations:** ^1^ Department of Gastrointestinal Surgery, Third Xiangya Hospital, Central South University, Changsha, Hunan, China; ^2^ Xiangya School of Medicine, Central South University, Changsha, Hunan, China; ^3^ Department of Pharmacy, Central South University, Changsha, Hunan, China

**Keywords:** colon adenocarcinoma, chromatin regulator, TCGA, prognostic, immune infiltration

## Abstract

Aberrant expression of chromatin regulators (CRs) could lead to the development of various diseases including cancer. However, the biological function and prognosis role of CRs in colon adenocarcinoma (COAD) remains unclear. We performed the clustering analyses for expression profiling of COAD downloaded from The Cancer Genome Atlas. We developed a chromatin regulator prognostic model, which was validated in an independent cohort data. Time-intendent receiver operating characteristics curve was used to evaluate predict ability of model. Univariate and multivariate cox regression were used to assess independence of risk score. Nomogram was established to assess individual risk. Gene ontology, and Kyoto Encyclopedia of genes and genomes, gene set variation analysis and gene set enrichment analysis were performed to explore the function of CRs. Immune infiltration and drug sensitivity were also performed to assess effect of CRs on treatment in COAD. COAD can be separated into two subtypes with different clinical characteristics and prognosis. The C2 had elevated immune infiltration levels and low tumor purity. Using 12 chromatin regulators, we developed and validated a prognostic model that can predict the overall survival of COAD patients. We built a risk score that can be an independent prognosis predictor of COAD. The nomogram score system achieved the best predict ability and were also confirmed by decision curve analysis. There were significantly different function and pathway enrichment, immune infiltration levels, and tumor mutation burden between high-risk and low-risk group. The external validation data also indicated that high-risk group had higher stable disease/progressive disease response rate and poorer prognosis than low-risk group. Besides, the signature genes included in the model could cause chemotherapy sensitivity to some small molecular compounds. Our integrative analyses for chromatin regulators could provide new insights for the risk management and individualized treatment in COAD.

## Introduction

In recent years, the morbidity and mortality of colon cancer have been increasing year by year, becoming one of the main causes of tumor-related death worldwide, which has caused a serious burden on people’s health and quality of life (Orangio, 2018; Ahmed, 2020). Metastasis and recurrence are the leading causes of death in most colon cancer patients (Labianca et al., 2010). At present, the main treatment for colon cancer is surgery, preoperative neoadjuvant chemoradiotherapy and postoperative chemoradiotherapy are the routine programs for comprehensive diagnosis and treatment of colon cancer (Wu, 2018). However, due to the insidious onset and asymptomatic progression of colon cancer, some patients with colon cancer are already in the middle and advanced stages when they are diagnosed, and conventional treatment cannot prolong the survival time of these patients (Freeman, 2013). Clinicians mainly assessed the prognosis of colon cancer patients by the disease process and tumor stage at the time of diagnosis (Pacal et al., 2020; Cerrito and Grassilli, 2021). However, traditional methods are insufficient to accurately assess the prognosis of colon cancer patients. Therefore, identifying biological markers related to colon cancer prognosis and survival is of great significance for patients with colon cancer.

Chromatin regulators (CRs) are a class of enzymes with specialized functional domains capable of recognizing, forming, and maintaining epigenetic states in a cellular context-dependent manner (Frye and Benitah, 2012). CRs are indispensable upstream regulators of epigenetics (Lam et al., 2005). According to their regulatory roles in epigenetics, CRs are generally classified into three major categories: deoxyribonucleic acid (DNA) methylation, histone modifications, and chromatin remodelers. Aberrant expression of CRs is associated with various biological processes such as inflammation, apoptosis, autophagy, and proliferation, suggesting that dysregulation of CRs may lead to the development of various diseases including cancer (Begolli et al., 2019; Smits et al., 2020; Lee and Kim, 2021). Therefore, CRs are expected to become new targets for the treatment of various diseases. However, the biological function and prognosis role of CRs in COAD remains unclear.

Many studies have shown that differences in tumor microenvironment, targets, and genes enhance the effects of traditional treatments and supplement the deficiencies of previous studies (Ansari et al., 2020; Lin et al., 2020). In the process of tumor progression, diagnosis, treatment and prognosis, bioinformatics has gradually played an important role with the continuous in-depth research of next-generation sequencing and big data centers (Jacoby et al., 2015). Through the analysis and comparative study of big data gene chip information, to calculate differential genes and immune-infiltrating cell screening in colon cancer to provide important biological prediction data for tumorigenesis mechanism and prognosis (Zhang et al., 2021). In our current research, we first explored the landscapes of chromatin regulators including differentially expressed genes, regulation network, correlations, and gene alterations in colon adenocarcinoma (COAD). Next, we performed the clustering analysis and identified the molecular subtypes and explored the characteristics of different subtypes. Then, we developed a prognostic model based on chromatin regulators in COAD, and validated the utility of this model in an independent cohort dataset, followed by the identification of an independent prognosis factors of risk score calculated by the chromatin regulators. Subsequently, we constructed a nomograph scoring tool for predicting the individual prognosis outcomes. Finally, we explored the pathways enrichment, immune filtration in different risk setting, evaluated the effect of chromatin regulators on immunotherapy in a cohort dataset, and identified the potential small molecular compounds associated with chemotherapy sensitivity. Our study highlights important role of chromatin regulators and provides new insights for individualized treatment in COAD.

## Materials and methods

### Data source

We downloaded the sequencing expression data of COAD from The Cancer Genome Atlas (TCGA: https://portal.gdc.cancer.gov/) including 473 tumor samples and 41 normal samples. We exclude these samples with mean absolute deviation (MAD)<0.1. The clinical information was also extracted, including age, gender, stage, TNM classification. The other independent dataset was also downed from the Gene Expression Omnibus database (https://www.ncbi.nlm.nih.gov/geo/) (GSE103479: 156 patients with colon carcinoma). The gene alterations and copy number variations were also obtained. We obtained the 870 chromatin regulators from the previous studies (Lu et al., 2018).

### Differential expression and gene alterations analysis

Using “limma” package, we identified the differentially expressed genes (DEGs) with the |log fold change|>1 and FDR *p* < 0.05. We built the protein-protein interaction network (PPI) using these DEGs in the STRING database (http://string-db.org), and these data were entered into Cytoscape Version 3.8 and generate the PPI network. We explored the correlation among these regulators using the Pearson correlation. Using “maftools” package, we analyzed the gene alterations and copy number variation in COAD.

### Identification of molecular subtypes

We first identified risk and favorable factors of the CRs using univariate cox regression. We performed the clustering analyses using “ConsensusClusterPlus” package, and identified the optimal the number of K using consensus matrix and consensus cumulative distribution function plot (Wilkerson and Hayes, 2010). Principle component analysis was used to validate the subtypes distributions. The Kaplan-Meier analysis was used to compare the survival curve between different subtypes. We explored the correlations of molecular subtypes with clinical characteristics using the Chi-square test.

To explore the differences in different subtypes, we calculated the enrichment score of each sample using this dataset: c2. cp.kegg.v7.4. symbols and performed the get set variation analysis (GSVA) using GSVA package (Hanzelmann et al., 2013). We also compared the immune status between two subtypes including estimate score, stromal score, immune score, and tumor purity. The infiltration levels of immune levels were also evaluated.

### Development and validation of prognostic model based on chromatin regulators

We first performed a univariate cox regression and identified the prognosis-related chromatin regulators (*p* < 0.001) in the TCGA training cohort. The least absolute shrinkage and selection operator (LASSO) regression was used to the identify the best genes number, followed by the multivariate cox regression to achieve the regression coefficient of the included genes in the model. We calculated the risk score of each sample according to the following formula: risk score = coef_1_*gene_1_ expression+…+coef_n_*gene_n_ expression. The COAD patients were divided into high-risk group and low-risk group according to the median of risk score. The Kaplan-Meier survival curves of different risk groups were plotted. We validated this established model using an independent cohort data (GSE103479). The time-intendent receiver operating characteristics curve (ROC) was plotted to calculate the area under the curve (AUC) at 1-year, 2-year, and 3-year in both TCGA training cohort and GEO validation cohort. PCA was also performed to identify the risk groups.

### Clinical characteristics and independent analysis

To investigate the correlations of risk groups with clinical characteristics, we compared the risk scores among different age (age>=60 vs. < 60), gender (male vs. female), stage (I-II vs. III-IV), T (T1-2 vs. T3-4), N (N0 vs. N1-2), M (M0 vs. M1) classification. We also showed the clinical characteristics and identified genes expression level between high-risk and low-risk groups. We further performed the univariate and multivariate cox regression to detect whether risk score could be an independent prognosis predictor of overall survival in COAD.

### Nomogram establishment and assessment

To estimate the individual’s prognosis risk, we built a nomogram score tool based on the following clinical characteristics: risk score, age, gender, stage, TNM classification. Using this nomogram tool, we can easily calculate the 1-year, 3-year, and 5-year overall survival (OS) rate. We plotted the calibration fitting line between observed OS and nomogram-predicted OS at 1-year, 3-year, and 5-year, which can assess the accuracy of nomogram.

Then, we calculated the AUCs of all clinical parameters, risk score and nomogram tool, and identified the predictive ability of nomogram tool. Decision curve analysis was used to determine the clinical practicability of nomograms based on the net benefit according to different threshold probabilities in COAD patients.

### Function enrichment and immune infiltration

To explore the biological function of different risk groups, we performed the gene setting enrichment analysis (GSEA) in high-risk group and low-risk group, respectively. We identified the top 5 signaling pathways of high-risk group and low-risk group. Then, we explored the immune infiltration status of high-risk and low-risk groups. We explored the correlations of risk score with immune cells by calculating the correlation coefficient. The tumor mutation burden level was also evaluated.

### Immunotherapy and chemotherapy sensitivity

To explore the effect of chromatin regulators on treatment, we first calculated the tumor immune dysfunction and exclusion level (Chen et al., 2022). Based on tumor pre-treatment expression profiles, this tumor immune dysfunction exclusion (TIDE) module can estimate multiple published transcriptomic biomarkers to predict patient response to immunotherapy. We also used the IMvigor210 cohort for validating the effect of CR regulators on immunotherapy, and the immunotherapy cohort data for urothelial carcinoma (Mariathasan et al., 2018). Using the CellMiner database, we further explored the chemotherapy sensitivity by calculating the Pearson correlation coefficients (Reinhold et al., 2012). |R|>0.25 and *p* < 0.05 were considered significantly correlated.

## Results

### Landscapes of chromatin regulator in colon adenocarcinoma

**FIGURE 1:**
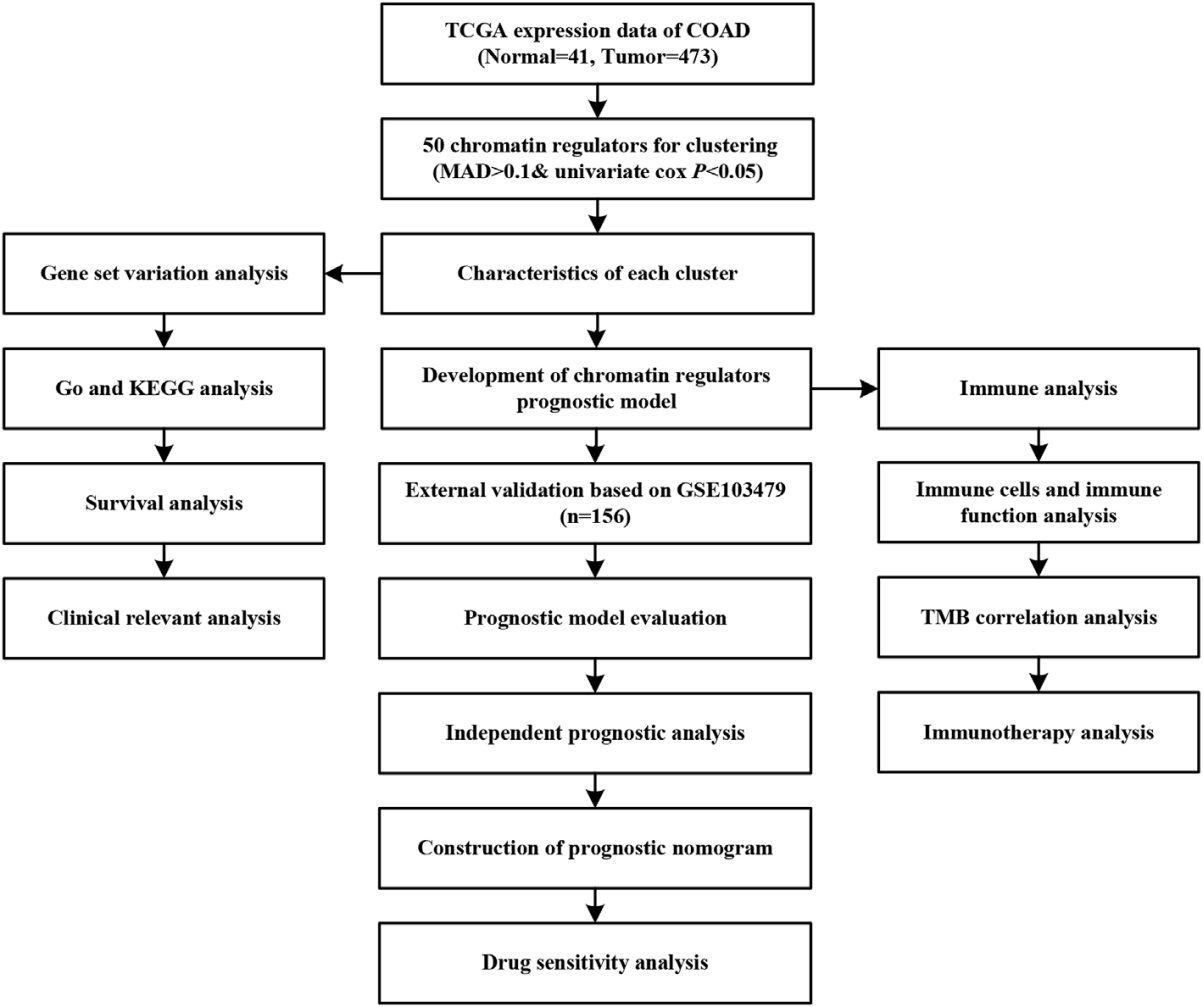
The flow chart of integrative analysis.

**FIGURE 2:**
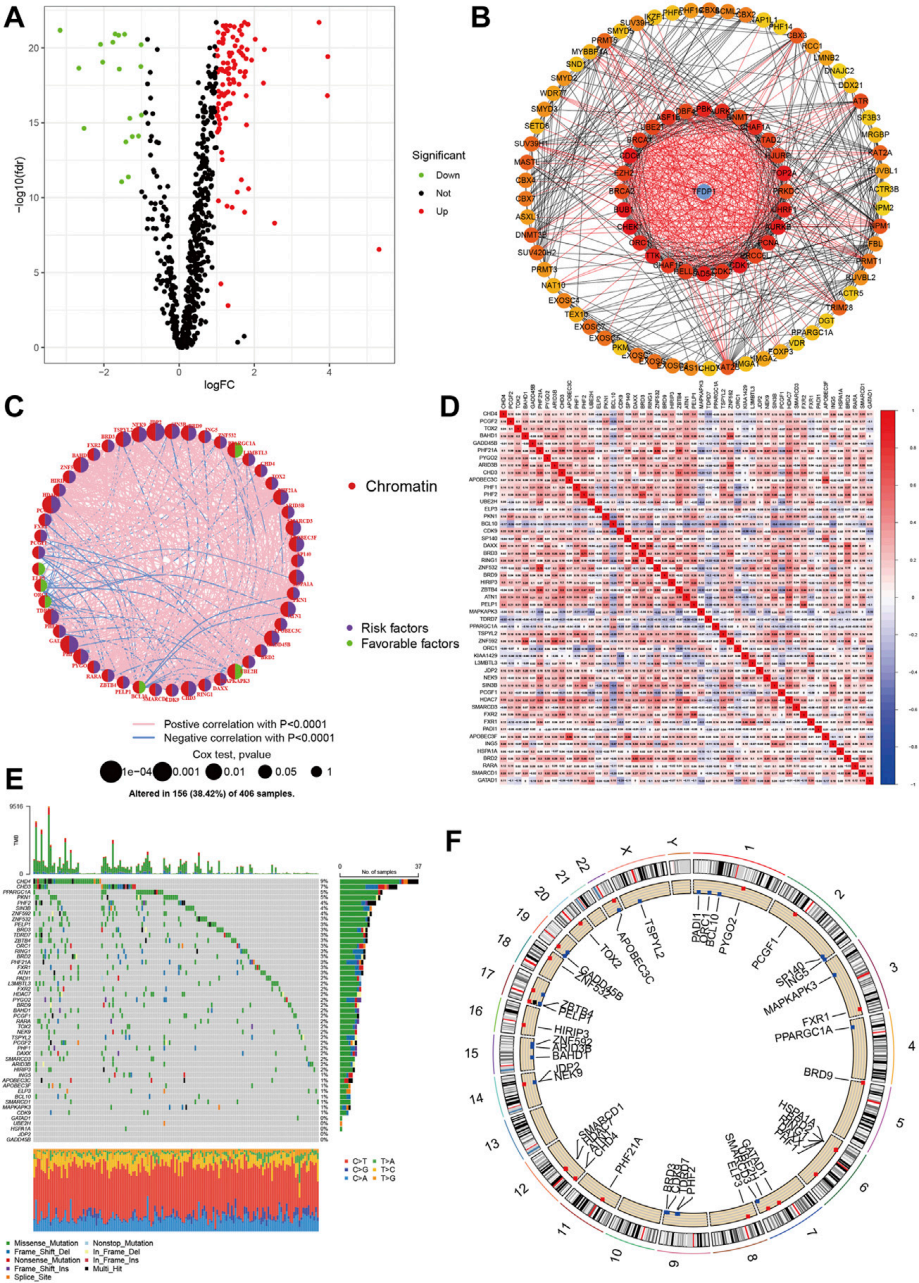
Landscapes of chromatin regulators in COAD. **(A)** Volcano showed the differential expression of chromatin regulators between tumor and normal samples. **(B)** Protein-protein interaction network of chromatin regulators. **(C)** Prognosis roles of chromatin regulators in COAD. **(D)** Heatmap showed the correlations among chromatin regulators. **(E,F)**: Gene alteration levels of chromatin regulators in COAD.

We depicted the landscapes of chromatin regulators in COAD using TCGA dataset. The flow of data processing was presented in Figure 1. We first performed differential expression analyses between tumor and normal samples using limma with |logFC|>1 and false discovery rate *p* < 0.05, and obtained 124 DGEs including 105 up-regulated genes and 19 down-regulated genes. The volcano plot presented the distributions of DGEs between tumor and normal samples (Figure 2A). Next, we built protein-protein interaction network and identified top 10 hub genes using normalized cross correlation methods (CHEK1, CDK1, TOP2A, CDC6, BUB1, AURK, TTK, RAD54L, PBK, UHRF1, Figure 2B). Among these chromatin regulators, we further identified 50 genes related to overall survival, including 6 favorable genes and 44 risky genes (Figure 2C). Then, we explored the correlations among these chromatin regulators, and found ZNF592-BAHD1 and ARID3B, PHF21A- ZBTB4 and ZNF532, BRD3-PHF2 and BRD2, APOBEC3F-APOBEC3C and SP140 showed strong positive correlations (*r* > 0.5) while BCL10 showed negative associations with other genes (Figure 2D). Finally, we analyzed the gene alterations of chromatin regulators in COAD. Our results indicated that the gene alterations ranged from 9% to 0% and top gene alterations of chromatin regulators in COAD were CHD4 (9%), CHD3 (7%), PPARGC1A (5%), PNK1 (5%), and PHF2 (4%) (Figure 2E). The C > T variations accounted for most of single nucleotide polymorphism in COAD. Figure 2F show the locations of gene mutations in chromosome.

### Identification of molecular subtypes

**FIGURE 3:**
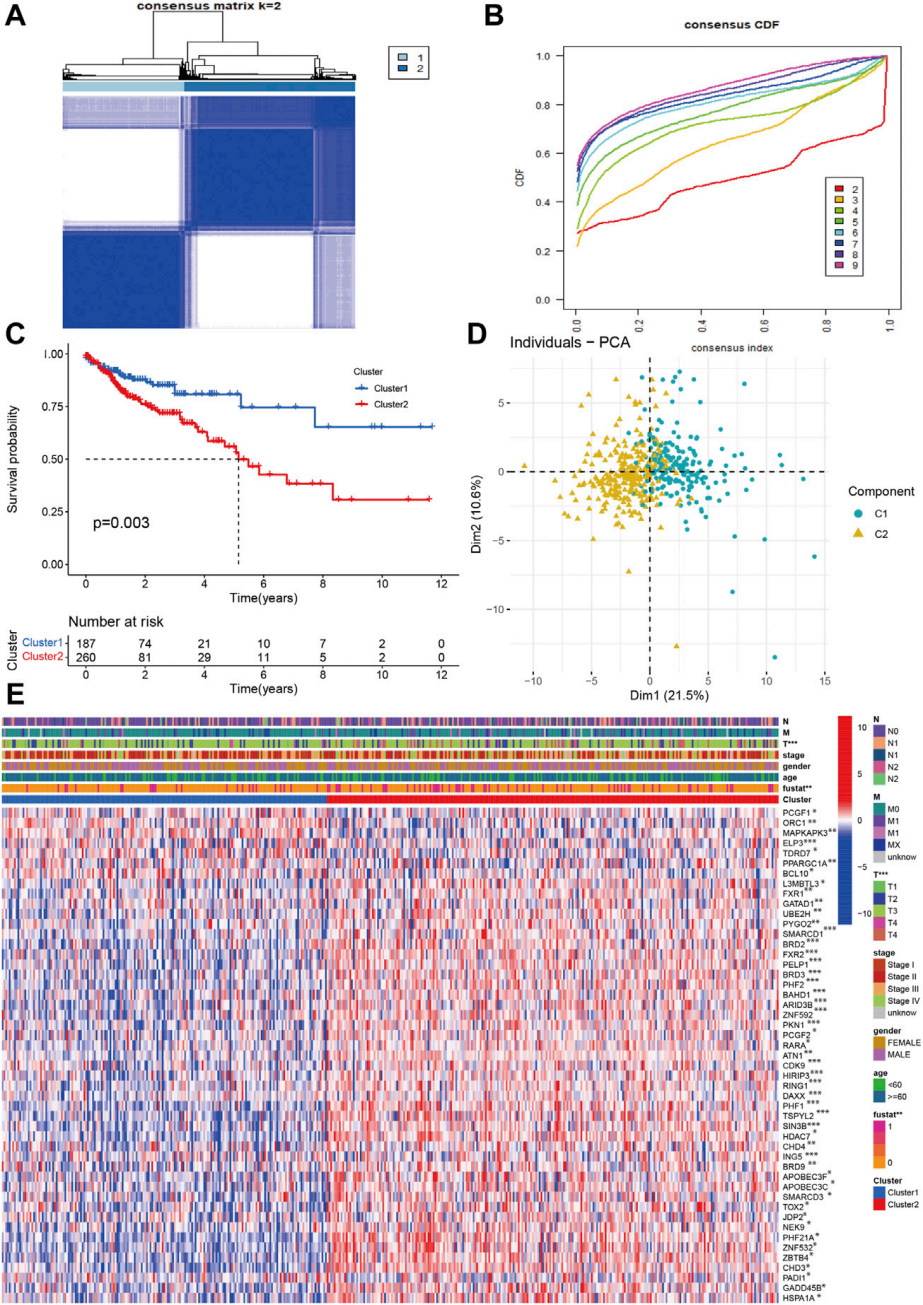
Identification of molecular subtypes based on chromatin regulators in OAD **(A,B)** The consensus matrix and CDF identified two subtypes in COAD. **(C)** The Kaplan-Meier survival curve of two subtypes. **(D)** PCA indicated two obvious components. **(E)** The correlations of molecular subtypes with clinical characteristics and gene expression profiling.

Using the chromatin regulators related to prognosis, we performed the consensus analysis. The consensus matrix showed that the optimal number is 2 (Figure 3A). The consensus CDF achieved the best values when the number of clustering was 2 (Figure 3B). The COAD can be divided into two subtypes (C1 = 187, C2 = 260). Then, the Kaplan-Meier analyses indicated that the C2 group had poorer prognosis than C1 group (*p* < 0.003, Figure 3C). The PCA also showed that COAD patients presented two distinguished two components. The Cluster 2 tend to be T III-IV stage (*p* < 0.01). There were no significant differences in age, gender, stage, N, M classification (Figures 3D,E). Some chromatin regulators were significantly down-regulated such as ORC1, MAPKAPK3, ELP3, TDRD7, and PPARGC1A.

**FIGURE 4:**
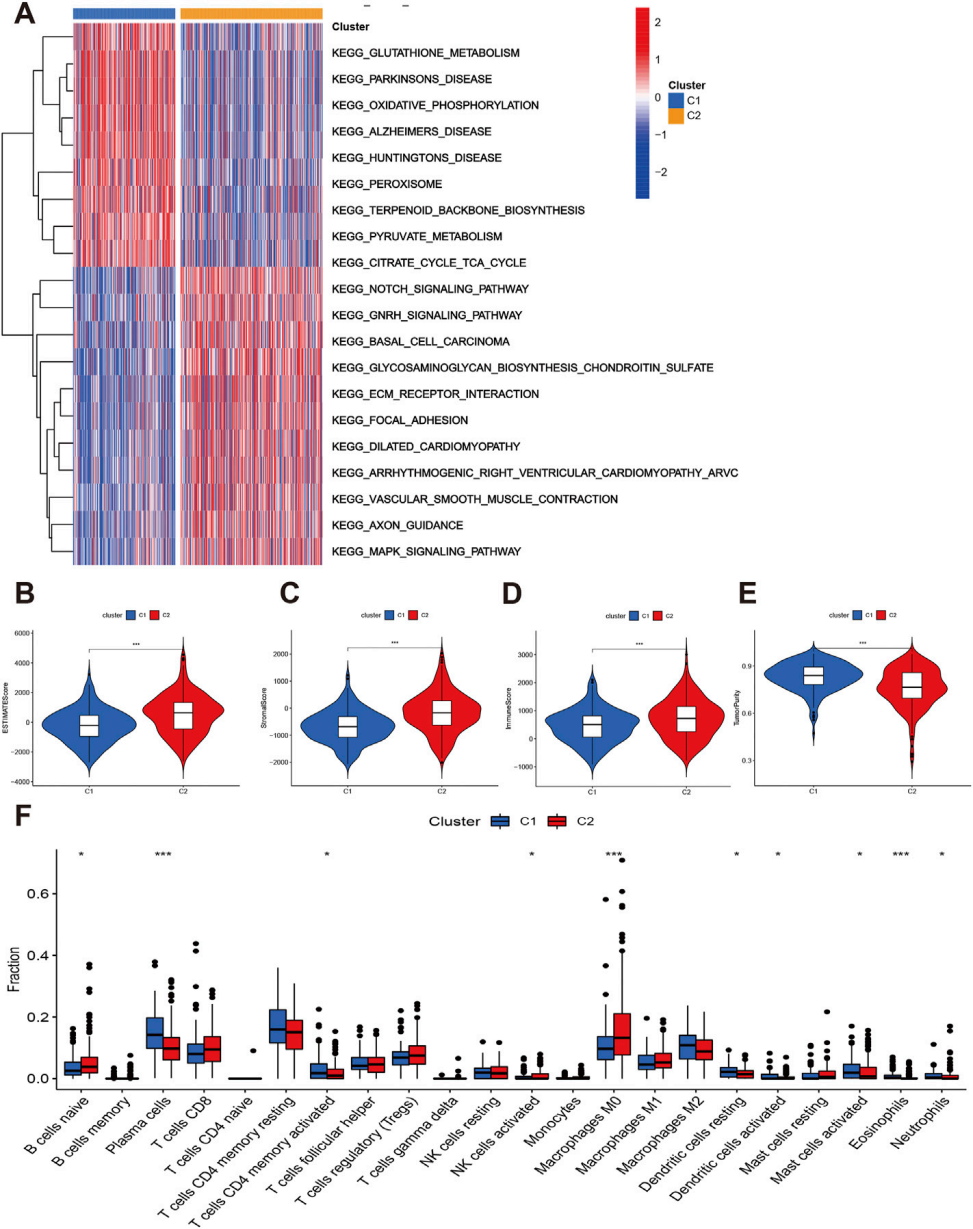
Function enrichment and immune status of two subtypes. **(A)** GSVA showed the differentially expressed signaling pathways. **(B–E)** Comparisons of immune infiltration levels between C1 and C2. **(F)** The infiltrations levels of immune cells between C1 and C2.

Furthermore, the GSVA indicated that some signaling pathways were significantly positive enriched in C2 such as Notch signaling pathways, GNRH signaling pathway, BASAL cell carcinoma, glycosaminoglycan biosynthesis chondroitin sulfate, ECM receptor interaction, focal adhesion, and MAPK signaling pathways. The glutathione and pyruvate metabolism, oxidative phosphorylation, peroxisome, terpenoid backbone biosynthesis, and citrate cycle tricarboxylic acid cycle were up-regulated in C1 group (Figure 4A).

Finally, we explored the immune infiltration status of two subtypes. The C2 had higher estimate, stromal and immune scores than C1 (Figures 4B–D). However, the tumor purity of C2 group was lower than C1 group (Figure 4E). The C2 group also have higher B cells naïve, NK cells activated, and macrophages M0 infiltration levels while the plasma cells, Tcells CD4 memory activated, dendritic cells activated, mast cells activated, eosinophils and neutrophils level of C1 group were significantly elevated (Figure 4F). We then considered the C2 group as “hot tumor” and C1 group as “cold tumor.”

### Development and validation of prognostic model based on chromatin regulators

**FIGURE 5:**
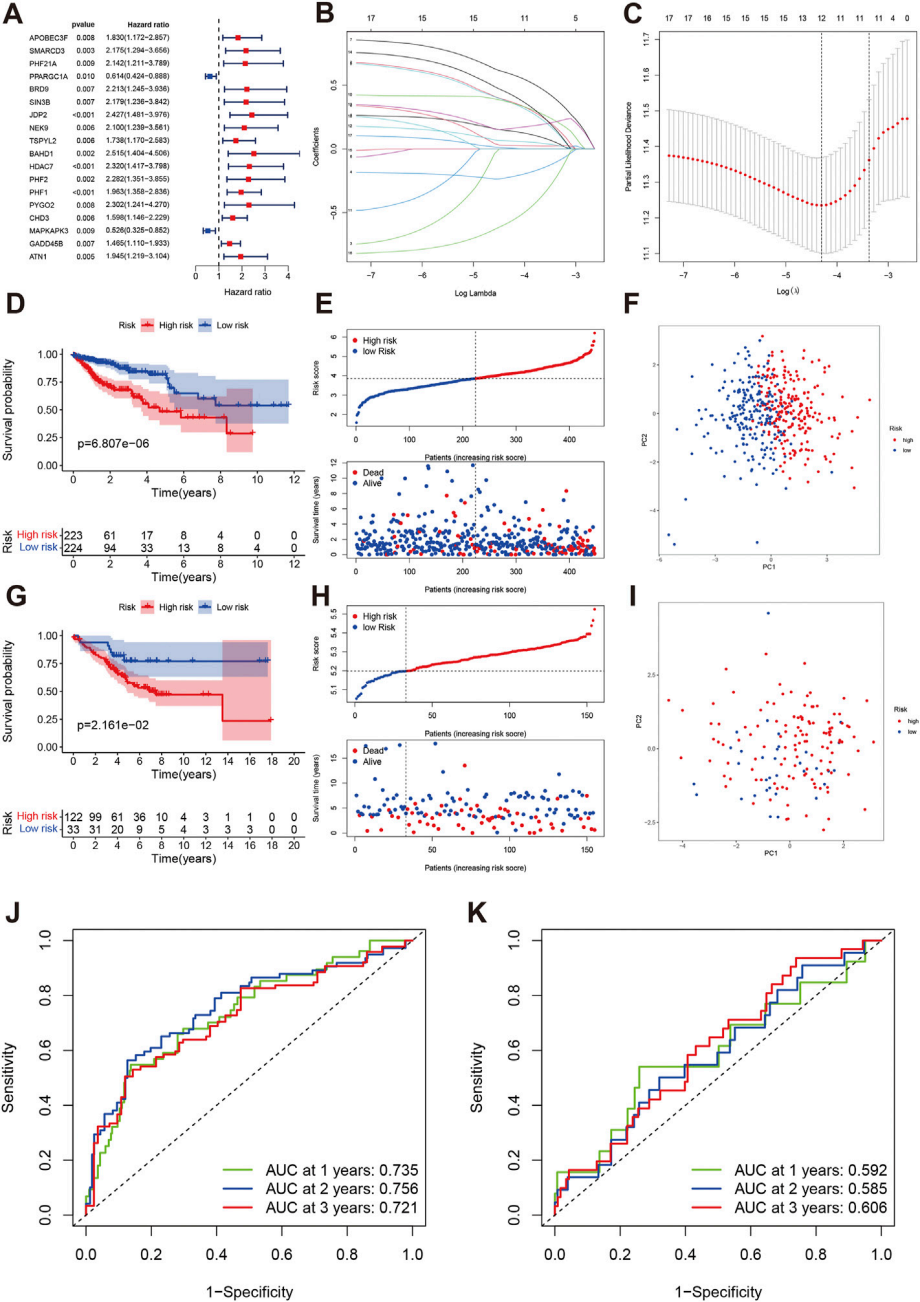
Development and validation of a chromatin regulator prognostic model in COAD. **(A)** The forest plot of univariate cox regression. **(B,C)** LASSO regression identified the number of genes included in the model. **(D–F)** The Kaplan-Meier survival curve, risk score and survival times distribution and PCA in TCAG training cohort. **(G–I)** The Kaplan-Meier survival curve, risk score and survival times distribution and PCA in GEO validation cohort. **(J)** ROC of risk score at 1-year, 2-year, 3-year in TCGA. **(K)** ROC of risk score at 1-year, 2-year, 3-year in GEO.

We first developed the prognostic model in TCGA training cohort. Using the FDR *p* < 0.01, we identified the 18 genes related to prognosis in COAD including two favorable genes (PPARGC1A and MAPKAPK3) and 16 risky genes (Figure 5A). We next performed the LASSO regression and identified the genes and number included in the prognostic model (Figures 5B,C). Twelve genes were included in the final model, and we established the following formula for calculating the risk score of each sample: risk score = EXPAPOBEC3F*0.142 + EXPSMARCD3 * 0.376 - PPARGC1A * 0.223 + BRD9*0.370 + JDP2*0.592 + NEK9 * 0.028 + BAHD1 * 0.366 + PHF2 * 0.063 + PHF1*0.158 + PYGO2*0.435 -MAPKAPK3 * 0.577 + GADD45B * 0.007. We divided the COAD patients into high-risk group (n = 223) and low-risk group (n = 224). The Kaplan-Meier analysis indicated that the high-risk group had worse overall survival than low-risk group (*p* < 0.001, Figures 5D,E). PCA also indicated two different risk groups (Figure 5F). Subsequently, we validated this model in an independent cohort data. Our results showed that the established model was well validated in this cohort (Figures 5G–I). The 1-year, 2-year, and 3-year AUCs were 0.735, 0.756, and 0.721 in the training cohort (Figure 5J). The AUCs were 0.592, 0.585, 0.606 at 1-year, 2-year, and 3-year, respectively (Figure 5K).

### Clinical correlations and independent analysis

**FIGURE 6:**
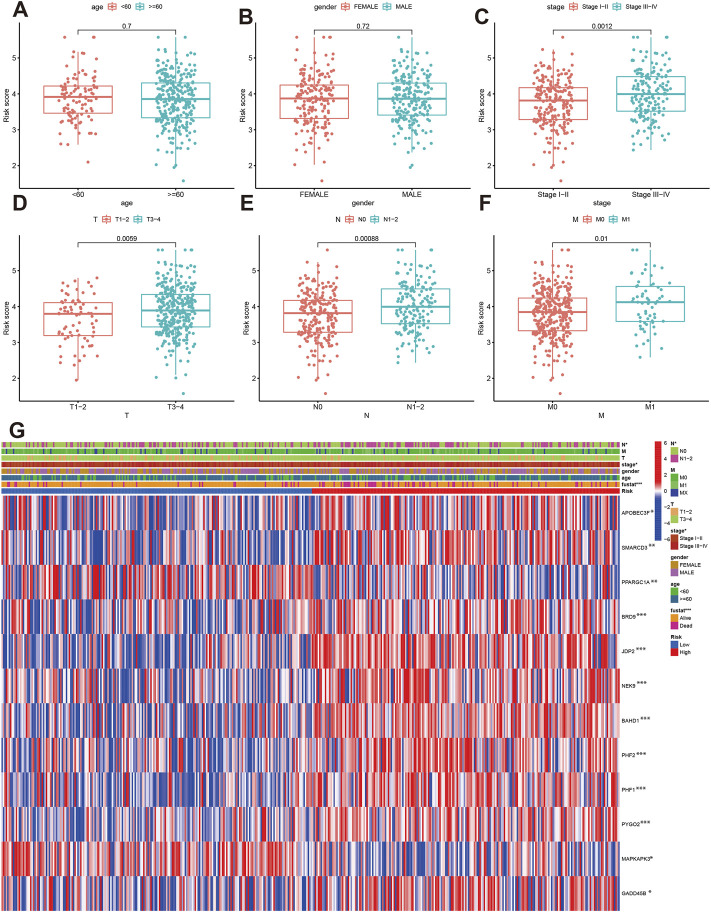
The correlation of risk score with clinical parameters. **(A–F)** Comparisons of risk score between different age, gender, stage, T, N and M classification. **(G)** The correlations of risk groups with clinical parameters and signature gene expression.

We further analyzed the correlations of risk score with clinical characteristics. The results indicated that age and gender were not associated with risk score (Figures 6A,B), while the patients with Stage III-IV, T3-4, N1-N2 and M1 had elevated risk score (Figures 6C–F). The high-risk group tend to be advanced clinical stage (Figure 6G). APOBEC3F, SMARCD3, BRD9, JDP2, NEK9, NAHD1, PHF2, PHF1, PYGO2, and GADD45B were significantly high-expressed in high-risk group.

**FIGURE 7:**
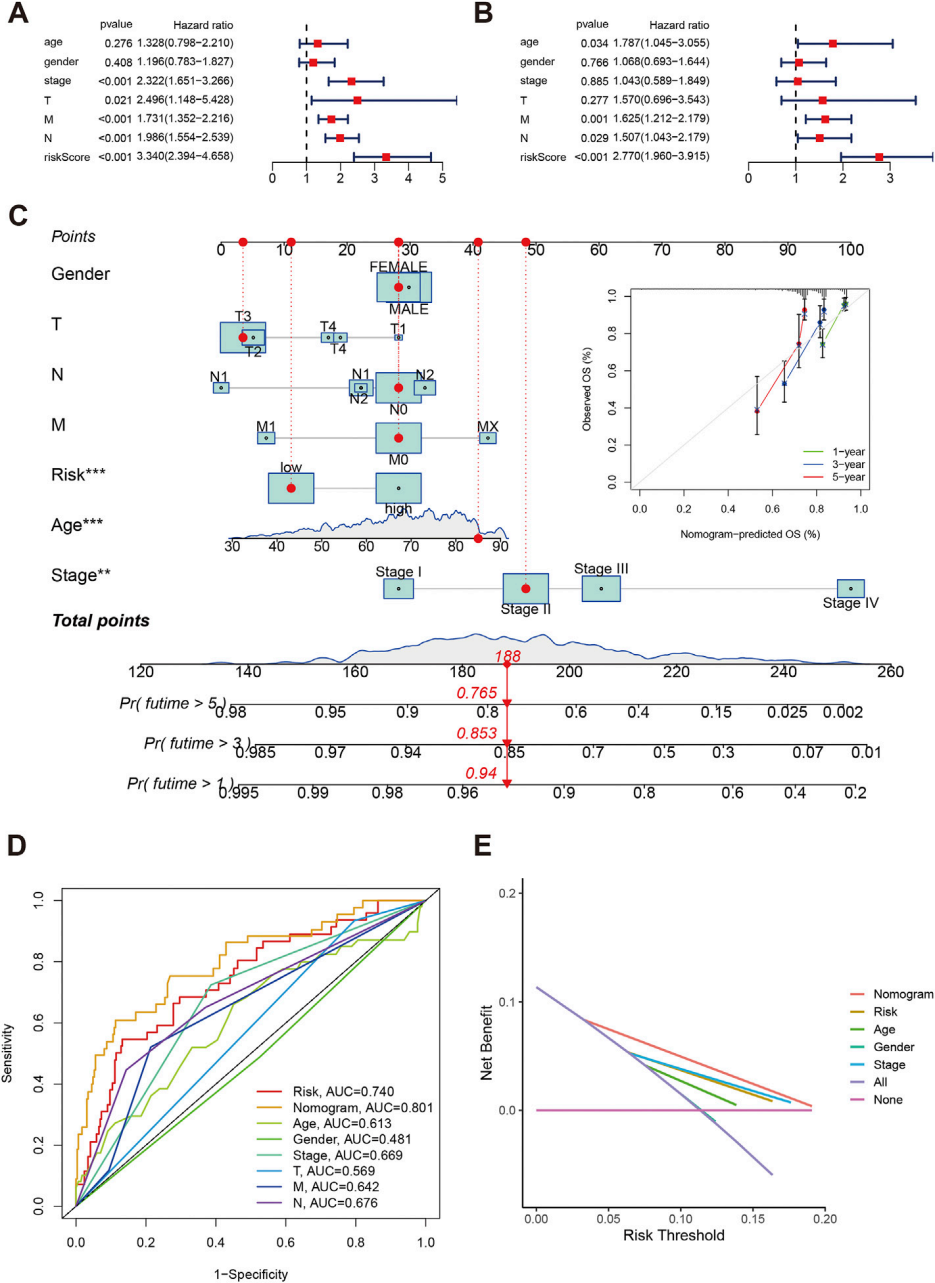
Independent analysis of risk score in COAD. **(A,B)** Forest plot of univariate and multivariate cox regression. **(C)** Nomogram and calibration plots using risk score and clinical characteristics. **(D)** Time-independent ROC of nomogram, risk score and clinical parameters. **(E)** Decision curve showed the clinical applications.

The univariate indicated that elevated risk score was significantly with poor overall survival (HR:3.34, 95%CI: 2.394–4.658, *p* < 0.001, Figure 7A), and the multivariate cox regression risk score is an independent prognosis predictor for COAD patients (HR:2.770, 95%CI: 1.960–3.915, *p* < 0.001, Figure 7B). Besides, Age, M1, and N1-2 classification were also risk factors for overall survival in COAD. Using clinical parameters and risk score, we built the nomogram score system (Figure 7C). We estimated the 1-year, 3-year and 5-year OS were 0.94, 0.853, and 0.765 for an 85-year male patient with T3, N1, low-risk, and Stage II. The calibrations plots of 1-year, 3-year and 5-year showed the nomogram-predicted OS and observed OS can be fitted well. Furthermore, the nomogram achieved the best predict ability (AUC = 0.801) followed by risk score (AUC = 0.740, Figure 7D). The decision curve analysis also indicated the nomogram can be well applied in the clinical practice because the nomogram has the best net benefit (Figure 7E).

### Function enrichment and immune infiltration

**FIGURE 8:**
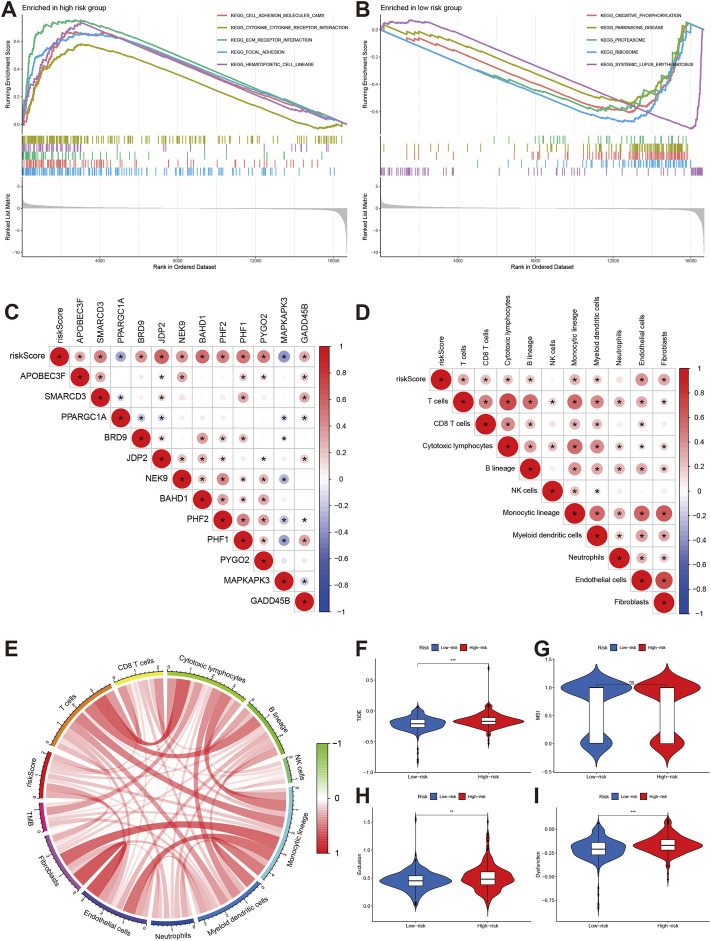
Pathway enrichment and immune infiltration of different risk groups. **(A,B)** KEGG pathways enrichment of high-risk and low-risk groups. **(C,D)** The correlations of risk score and signature gene and immune cells. **(E)** correlations of risk score with TMB. **(F–I)** Comparisons of TIDE, MSI, exclusion and dysfunction between high-risk group and low-risk groups.

The GSEA indicated the top 5 enrichments were cell adhesion molecules cams, cytokine receptor interaction, extracellular matrix receptor interaction, focal adhesion, and hematopoiesis cell lineage in high-risk group (Figure 8A), while the top 5 enrichments were oxidative phosphorylation, Parkinson’s disease, proteasome, ribosome, and systemic lupus erythematosus in low-risk group (Figure 8B). The risk score was positively associated with APOBEC3F, SMARCD3, BFRD9, JDP2, NEK9, BAHD1, PHF1, PHF2, PYGO2, and GADD45B. the PPARGC1A and MAPKAPK3 were negatively associated with risk score (Figure 8C).

The immune infiltration analyses indicated that risk score was positively associated with T cells, CD8T cell, cytotoxic lymphocytes, B lineage, monocytic lineage, myeloid dendritic cells, endothelial cells, and fibroblasts (Figure 8D). The risk score was also positively related to tumor mutation burden (TMB) level (Figure 8E).

### Immunotherapy and chemotherapy sensitivity

**FIGURE 9:**
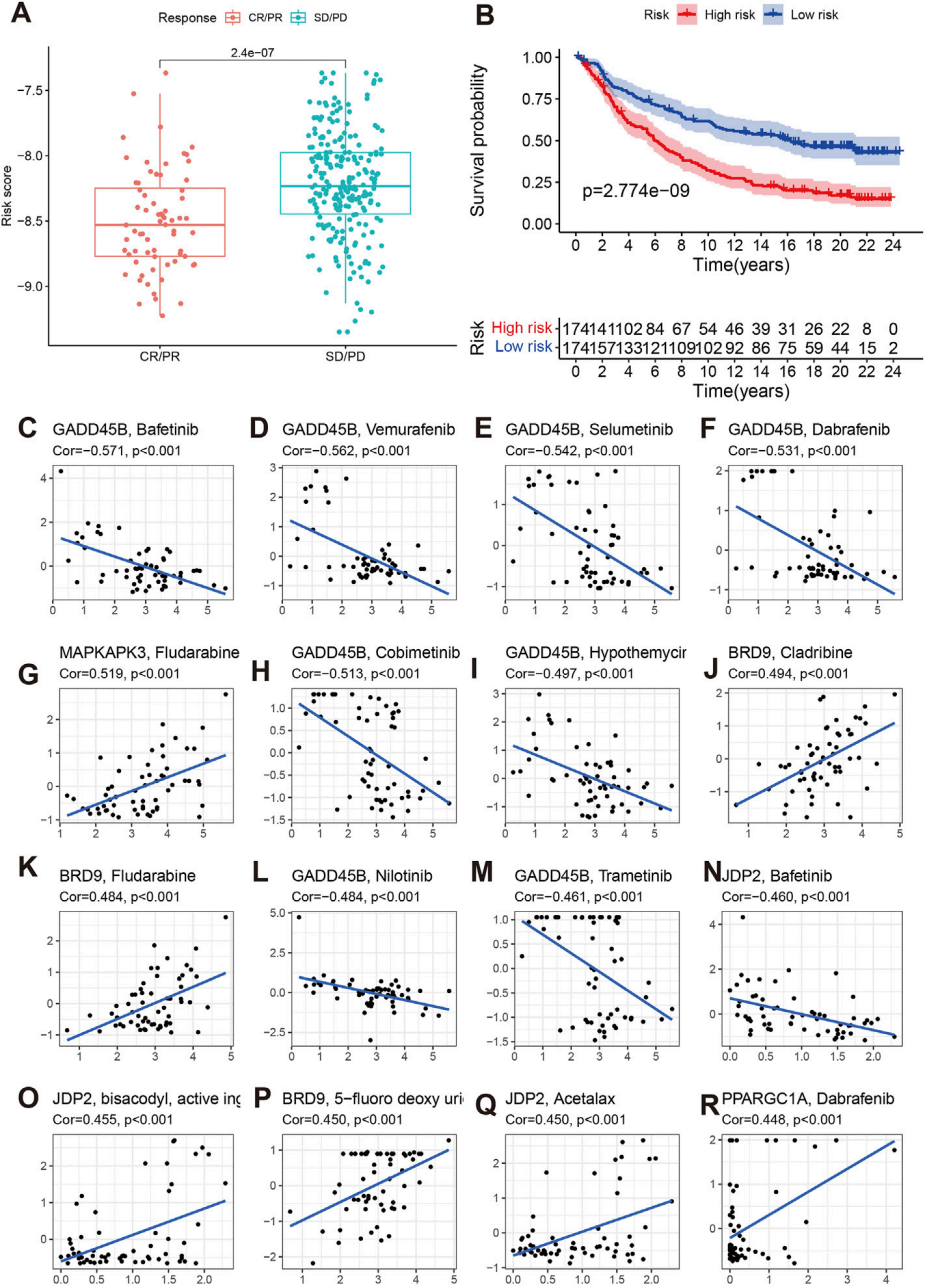
Effect of chromatin regulators on immunotherapy of IMvigor data **(A,B)** of and chemotherapy sensitivity **(C–R)**.

We also explored the effect of chromatin regulators on immunotherapy. We first evaluated the tumor immune dysfunction and exclusion level (TIDE). Our results indicated that the high-risk group had higher TIDE, exclusion, and dysfunction levels except MSI (Figures 8F–I), which means the high-risk group had poor response to immunotherapy. The IMvigor data confirmed our results. The stable/progression disease group had higher risk score than the com complete/part remission (Figure 9A). Furthermore, the high-risk had poorer overall survival than low-risk group (Figure 9B, *p* < 0.001).

We then evaluated the effects of signature genes on chemotherapy sensitivity. We found that GADD45B can cause chemotherapy resistance to Bafetinib, Vmurafenib, Selumetinib, Dabrafenib, Cobimetinib, Hypothemycir, Trametinib, and Nilotinib. MAPKAPK3, BRD9, JDP2, PPARGC1A can enhanced the sensitivity of some small molecular compounds, including Fludarabine, Cladribine, 5-fluoro deoxy urine uracil, Acetalax, and Dabrafenib (Figures 9C–R).

## Discussion

The present study has the following several findings (Ahmed, 2020): The COAD can be separated into two subtypes with clinical characteristics, prognosis outcomes, and biological function enrichment. The C2 group had elevated immune infiltration levels and low tumor purity, which can be considered as “hot tumor” and the C1 group had low level immune status considered as “cold tumor” (Orangio, 2018). Using 12 chromatin regulators, we developed a prognostic model that can predict the overall survival and risk classifications among COAD patients. This model was well validated in an independent external cohort data (Labianca et al., 2010). We built a risk score that can be an independent prognosis predictor of COAD. The high-risk group based on risk score tended to have risky clinical characteristics (Wu, 2018). Using clinical parameters and risk score, we built the nomogram score system that can achieve the best predict ability and were also confirmed by decision curve analysis about its clinical application (Freeman, 2013). There were significantly different function and pathway enrichment, immune infiltration levels, and TMB level between high-risk and low-risk group (Pacal et al., 2020). The high-risk group had poor response to immunotherapy. The external validation data also indicated that high-risk group had higher SD/PD response rate and poorer prognosis than low-risk group. Besides, the signature genes included in the model could cause chemotherapy sensitivity to some small molecular compounds. Our integrative analyses for chromatin regulators could provide new insights for the risk management and individualized treatment in COAD.

Epigenetic changes, considered to be one of the most important markers of tumors, are driven by chromatin regulator (Dey, 2011; Florea and Karaoulani, 2018). The chromatin regulators dynamically regulate chromatin structure and epigenetic regulation of gene expression in response to endogenous and exogenous signaling cues (Weaver and Bartolomei, 2014). Somatic changes or misexpression of CR may reprogram the epigenetic map of chromatin, leading to a wide range of common diseases, especially cancer (Shu et al., 2012). Currently, the function role of chromatin regulators in COAD is still unclear. We first explored the relevance in prognosis and treatment for COAD. We identified two molecular subtypes using prognosis-related chromatin regulators. Two subtypes had different expression profiling of chromatin regulators and clinical characteristics. The cluster 2 showed elevated stromal and immune activation and was mainly enriched in some important tumor-related signaling pathways such as Notch, Gnrh, and MAPK signaling pathways, which had been suggested to be closely associated with tumor occurrences (Kranenburg, 2015; Lajko et al., 2019; Tang et al., 2021). ECM receptor interaction and focal adhesion were also highly enriched in cluster 2. On the contrary, the cluster 1 had low immune infiltration level and was mainly enriched in some metabolism-related pathways and functions such as glutathione, pyruvate, TCA cycle and oxidative phosphorylation. Thus, the cluster 2 can be regarded as “hot tumor,” and the cluster 1 was called “cold tumor.” Whether the tumor is hot or cold affects whether immunotherapy, represented by PD-1 inhibitors, is effective. This is because tumor cells overexpress PD-L1 protein and induce high expression of PD-1 on immune cells such as T lymphocytes. When the two are combined, they inhibit the function of T lymphocytes, allowing tumors to escape immune attack (Reschke and Olson, 2022).

Using these chromatin regulators, we established a prognostic model with twelve chromatin regulators. Previous studies also established prognostic models using other gene sets. Zhou et al. developed an autophagy-related lncRNA model for COAD and the 3-year predictive AUC was 0.790, which was close to our model (Zhou et al., 2020). Using 44 ferroptosis-related lncRNAs, Li developed a prognostic model with AUC of 0.860 that was slightly higher than our AUC (Li et al., 2021). Li also built a prognostic model using immune-related genes, and the predictive ability was 0.792. Broadly speaking, all these model had similar predictive abilities, which suggested that our model was effective (Miao et al., 2020).

In this model, PPARGC1A and MAPKAPK3 were favorable genes in this model. Previous study had reported that the expression of PPARGC1A was negatively associated with some immune cells, which means that PPARGC1A may be responsible for regulating the immune components of tumor microenvironment (Ma et al., 2021). As a member of the Ser/Thr protein kinase family. MAPKAPK3 functions as a mitogen-activated protein kinase (MAP kinase)- activated protein kinase. Previous studies reported that ERK, p38 MAP kinase and Jun N-terminal kinase were all able to phosphorylate and activate this kinase, which suggested the role of this kinase as an integrative element of signaling in both mitogen and stress responses (Wagner and Nebreda, 2009; Sun et al., 2015). It was reported that MAPKAPK3 can promote autophagy via some phosphorylation pathway *in vivo* and vitro, which may explain its favorable role in COAD (Wei et al., 2015). The other 10 gene were oncogenes in the model. Such as APOBEC3F that could be a new treatment target in multiple cancers including COAD (Svoboda et al., 2016). SMARCD3 (Jiang et al., 2020), BRD9 (Sabnis, 2021), JDP2 (Mansour et al., 2018), were also reported to be a oncogene role in some cancer. We calculated the risk score for each sample based on the established prognostic model and divided COAD patients into high-risk and low-risk groups. The high-risk group and low-risk group had different overall survival. The time-independent ROC indicated that the prognostic signature with 12 chromatin regulators had accurate and reliable predictive ability. The established model was effectively validated in an independent cohort data. The univariate and multivariate cox regression also demonstrated that risk score was an independent risk factor for poor overall survival. Based on the risk score and clinical parameters, we constructed a nomogram scoring tool for individual’ survival outcomes. The calibration, ROC and decision curve analysis had excellent predictive ability.

The risk score was found to be positively associated with many immune cells including T cells, CD8 T cells, monocytic lineage, endothelia cells and fibroblasts. We also found that the high-risk group and low-risk group had different immune infiltration levels. Immune cell infiltration in tumor microenvironment affects the prognosis of tumor therapy (Bader et al., 2020; Lei et al., 2020). To explored the effect of chromatin regulators on immunotherapy, we further evaluated the TIDE levels of different risk groups. We found that the high-risk group had relatively high immune status including TIDE, exclusion, and dysfunction, which means the high-risk group may have poor prognosis when receiving immunotherapy. The data from an immunotherapy cohort data (IMvigor210) confirmed these assumptions that patients with high-risk score and immune infiltration had poor prognosis (Vander et al., 2019). Recently, several clinical trials had been performed to explore the efficacy of immunotherapy (Bao et al., 2020; Mlecnik et al., 2020). Our results provided some references for these researches. Finally, we evaluated the effect of chromatin regulators on chemotherapy sensitivity, and found GADD45B can cause chemotherapy resistance to Bafetinib, Vmurafenib, Selumetinib, Dabrafenib, Cobimetinib, Hypothemycir, Trametinib, and Nilotinib. MAPKAPK3, BRD9, JDP2, PPARGC1A can enhanced the sensitivity of some small molecular compounds, including Fludarabine, Cladribine, 5-fluoro deoxy urine uracil, Acetalax, and Dabrafenib. These findings will help clinical treatment for COAD patients.

The present study had several limitations. First, the sample size of validation cohort was small, and study with larger sample size were required. Based on suggestions from professional filed, at least two independent cohorts were required for the present prognostic model. Second, the biological function, molecular mechanism and the effect of chromatin regulators were not validated through experiments *in vivo* and vitro. Data from experimental research will further refine the present findings. Although we evaluated the effect of chromatin regulators on immunotherapy using two different methods, the immunotherapy was carried out in the other tumor types. Studies performed in COAD will be more persuasive.

In conclusion, we obtained two molecular subtypes in COAD using chromatin regulators, which had different clinical characteristics and immune landscapes. We further established and validated a chromatin-related prognostic model that can be capable of predicting overall survival of COAD patients. More important, we also found that chromatin regulators could affect the immunotherapy and chemotherapy sensitivity in COAD patients. Our study will provide new risk management and individualized treatment strategies for COAD that could bring more benefits for patients.

## Data Availability

Publicly available datasets were analyzed in this study. TCGA dataset was from the Cancer Genome Atlas (https://portal.gdc.cancer.gov/), and GSE10379 were from Gene Expression Omnibus database (https://www.ncbi.nlm.nih.gov/geo/).
